# Supplementary material for: Synaptic Targets of Glycinergic Neurons in Laminae I–III of the Spinal Dorsal Horn
Source: Int J Mol Sci. 2023 Apr 8;24(8):6943. doi: 10.3390/ijms24086943 (PMC10139066; doi:10.3390/ijms24086943)
Supplement: Supplementary file 1 [file ijms-24-06943-s001.zip › ijms-2312699-supplementary.pdf]

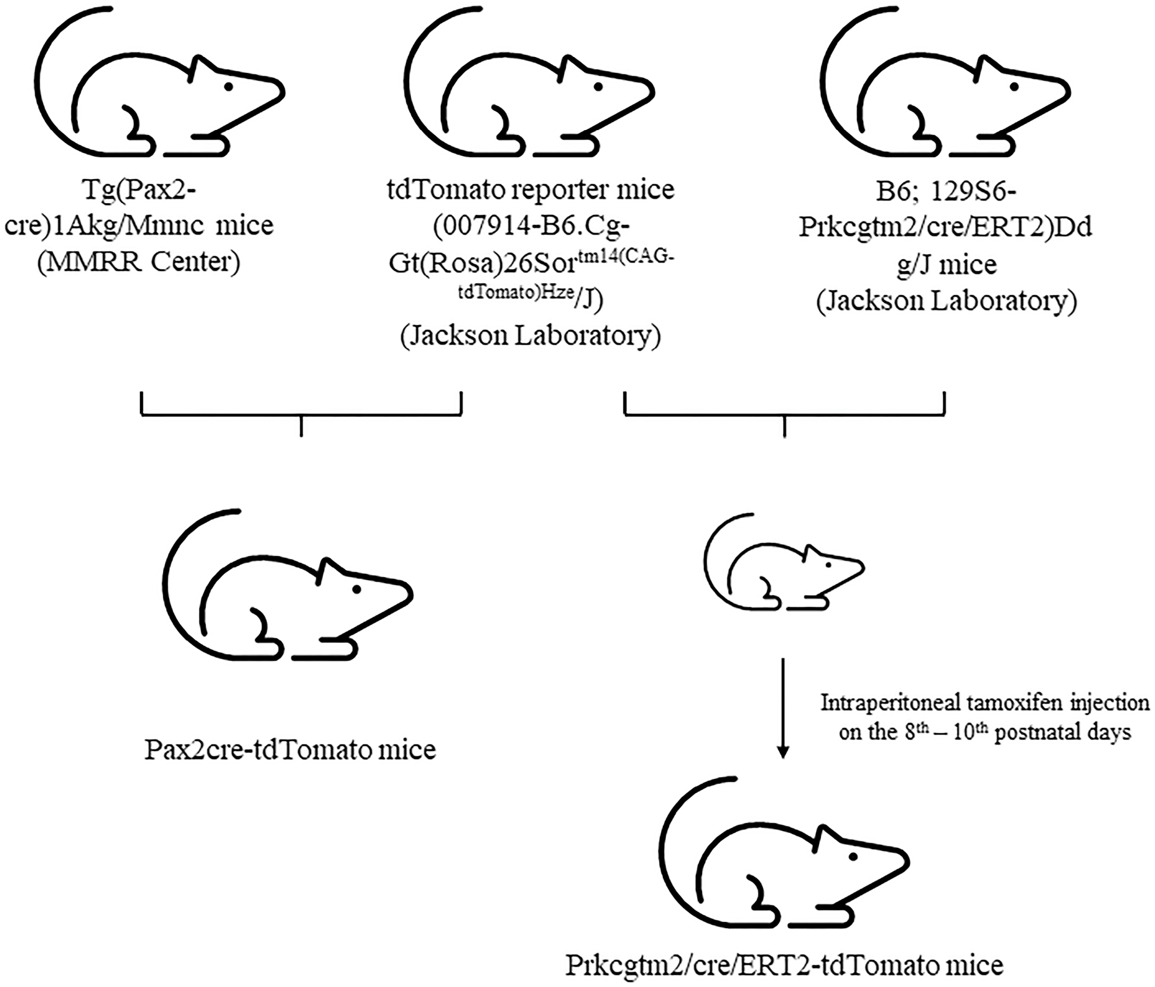

Supplementary Figure S1. Schematic representation showing how the transgenic animals were crossed and treated with tamoxifen to induce tdTomato expression in the genetically modified neurons.
